# Changes in sexual desires and behaviours of people living with HIV after initiation of ART: Implications for HIV prevention and health promotion

**DOI:** 10.1186/1471-2458-11-633

**Published:** 2011-08-08

**Authors:** Joyce Wamoyi, Martin Mbonye, Janet Seeley, Josephine Birungi, Shabbar Jaffar

**Affiliations:** 1 National Institute for Medical research, P.O Box 1462, Mwanza, Tanzania; 2 MRC/UVRI Uganda Research Unit on AIDS, P.O. Box 49, Entebbe, Uganda; 3 School of International Development, University of East Anglia, Norwich, NR4 7JT, UK; 4 The AIDS Support Organisation, Kampala, Uganda; 5 London School of Hygiene and Tropical Medicine, Keppel Street, London WC1E 7HT, UK

**Keywords:** Sexual desire, ART, HIV, Longitudinal, Sexual behaviour

## Abstract

**Background:**

As immune compromised HIV sero-positive people regain health after initiating antiretroviral treatment (ART), they may seek a return to an active 'normal' life, including sexual activity. The aim of the paper is to explore the changing sexual desires and behaviour of people on ART in Uganda over a 30 month period.

**Methods:**

This study employed longitudinal qualitative interviews with forty people starting ART. The participants received their ART, adherence education and counselling support from The AIDS Support Organisation (TASO). The participants were selected sequentially as they started ART, stratified by sex, ART delivery mode (clinic or home-based) and HIV progression stage (early or advanced) and interviewed at enrolment, 3, 6, 18 and 30 months of their ART use.

**Results:**

Sexual desire changed over time with many reporting diminished desire at 3 and 6 months on ART compared to 18 and 30 months of use. The reasons for remaining abstinent included fear of superinfection or infecting others, fear that engaging in sex would awaken the virus and weaken them and a desire to adhere to the counsellors' health advice to remain abstinent. The motivations for resumption of sexual activity were: for companionship, to obtain material support, social norms around marriage, desire to bear children as well as to satisfy sexual desires. The challenges for most of the participants were using condoms consistently and finding a suitable sexual partner (preferably someone with a similar HIV serostatus) who could agree to have a sexual relationship with them and provide for their material needs.

**Conclusions:**

These findings point to the importance of tailoring counselling messages to the changing realities of the ART users' cultural expectations around child bearing, marriage and sexual desire. People taking ART require support so they feel comfortable to disclose their HIV status to sexual partners.

## Background

Antiretroviral treatment (ART) has reduced mortality and morbidity from HIV disease improving the wellbeing of many people living with HIV [1-4]. The perceived threat of HIV has decreased with increased HIV treatment optimism and beliefs that HIV treatment eliminates the risk for HIV transmission [5-7]. It is not surprising that as people regain health, and return to their productive lives, they resume sexual activity [6,8].

There are concerns that increased use of ART may be associated with increased sexual risk taking [5,8-10]. As ART becomes increasingly available in many African communities it is important to understand the effects on the sexual desires and behaviours of users as this has implications for the spread and control of the HIV epidemic.

Although studies have been conducted on the sexual behaviour of people on ART, most have focused on sexual partnerships, sexual acts and the consequences of risky sexual behaviour [6,7,9,11]. There is a dearth of studies employing a longitudinal qualitative design to understand the evolving experiences and sexual desires of people on ART as they progress on medication. In this paper we describe the changing sexual desires and behaviour over a 30 month period of 40 people initiating ART in Uganda. Valuable insights into potential areas of support, including appropriate counselling messages, for people living with HIV and on ART can be gained from understanding how the sexual desires and behaviour develop after treatment initiation and the strategies adopted to find sexual partners to satisfy their desires.

## Methods

### Study population and setting

Participants were recruited from the Jinja branch of The AIDS Support Organization (TASO), a non-governmental organisation that provides services for people living with HIV in Uganda. TASO delivers client centred counselling sessions including advice on: seeking treatment promptly, avoiding HIV superinfection, prevention of HIV transmission, and reduced sexual activity. It also provides treatment to clients and their families as well as offering voluntary counselling and testing to other family members. TASO also provides non-spermicidal male condoms to all clients among other items in their basic care package, which help the client live with their infection [12].

### Procedure for sampling

The qualitative study complemented a cluster-randomised equivalence trial of care strategies with 1453 participants, who were recruited when they started ART. The trial compared the effectiveness of a home-based care service utilising lay health workers with a health facility-based approach [13]. We planned to enrol a total of 40 participants (20 males and 20 females) for this qualitative study based on where they received ART (health facility - 20 or at home - 20). One male participant from the home arm died after the enrolment interview and was replaced bringing the total enrolment sample to 41. We aimed to compare the adherence levels of two immunologically different groups: half of the sample were clients who were starting ART with CD4 counts of between 150 and 200 (high) while the other half had CD4 counts below 100 (low).

### Data collection

Data collection included in-depth interviews and home observation visits using a checklist. While in-depth interviews were conducted to explore the personal experience of ART users' over time, observation was mainly to provide contextual data for deepening the understanding of environmental and social influences. All enrolment interviews were conducted at the TASO health facility. At enrolment, participants were asked for permission to conduct further interviews from their homes; all participants agreed to this. Therefore the three month interviews and subsequent follow-up interviews were almost always conducted in the homes of the participants. Home observations were recorded as field notes which added to the contextual data. Almost all interviews were conducted in the widely used language (Luganda). Interviews were done by three trained and experienced interviewers (one male and one female) who were familiar with the area and study methods. Recruitment was done between October 2005 and April 2006. Sequential recruitment did not allow for matching interviewers and respondents by gender. Each participant stayed with the same interviewer throughout the study and this partly explained the strong rapport which grew with each round of interviewing.

To obtain information on their experience of ART and changes in their sexual behaviour over time, participants were interviewed at enrolment then at 3, 6, 18, and finally at 30 months on ART. Some (n = 7) of the participants died before they completed all the interview rounds. As indicated in Table 1, out of the 41 participants (20 females, 21 males) enrolled in the study, not all participants were available for interview during all the four rounds. The participant who refused to be interviewed simply exercised his right not to participate. He did not give any reason why he had opted out.

**Table 1:** Changes in availability of participants for interview at different time periods on ART

Period on ART	Men	Women
Enrolment	21 interviewed (1 to replace deceased one)	20 interviewed
3 months	16 interviewed (4 not interviewed because inaccessible, 1 deceased)	14 interviewed (1 deceased before interview, 5 inaccessible)
6 months	20 interviewed (1 deceased)	14 interviewed (3 deceased, 3 inaccessible)
18 months	18 interviewed (3 deceased)	17 interviewed (3 deceased)
30 months	17 interviews (3 deceased, 1 refusal)	16 interviewed (4 deceased)

The interview topics included sexual desire before and after commencement of ART, attitudes towards sex and condom use, fertility intentions, adherence to ART, disclosure of HIV status and concerns about HIV transmission. The interviews lasted between one and two hours and were tape recorded with permission from respondents.

To protect confidentiality, a coding system was devised to refer to each participant, with the first letter referring to sex (M for male and F for female), the second to drug delivery mode (H for home and F for facility) and the third to early or advanced HIV stage at enrolment (H for high CD4 count or early HIV progression or stage and L for low CD4 count or advanced HIV progression). The clients were also numbered from 1 to 5 within each stratum (or 6 for the stratum including the participant who died). This coding system has been used when referring to participants in this paper.

The study was approved by the Uganda National Council for Science and Technology, the Science and Ethics Committee of the Uganda Virus Research Institute, Ethics boards of the US Centres for Disease Control and Prevention, and London School of Hygiene and Tropical Medicine. All participants gave written informed consent before enrolment into the study.

### Analysis

The data were transcribed and translated to English, entered into QSR NVIVO 8 software and coded. Constant comparative techniques were used to analyse the data (Strauss & Corbin, 1998). Analysis began with an open coding approach, using the participants' language, avoiding where possible the imposition of preconceived theoretical constructs. Based on the data from the first 10 interviews (5 males, 5 females), an initial set of "open codes" was developed. Examples of open codes were: "sexual negotiation", "partner types" and "disclosure of HIV status to partners", "disclosure of HIV status to non-partners", "sexual attractiveness", "duration of sexual relationships", "knowledge and beliefs about ART", "fertility intentions", sexual exchange, and "condom use". During this first stage of analysis, "open codes were assigned to segments of the transcript. As additional interviews were completed, axial codes were used to group the open codes into more abstract conceptual categories. Examples of axial codes were "motivations for having sex", "attitudes towards sex" and "finding sexual partners and disclosure of HIV status". As the analysis continued, we began to see how the sexual desires of people on ART changed over time with progress of their return to health. At this point we began to incorporate the individual participants' experiences with their broader descriptions.

During the final stage, attention was paid at how the individual experiences reflected the broader social-cultural beliefs and expectations on issues such as marriage and child bearing and having HIV. This stage of analysis integrated previous findings from theoretical and empirical literature to inform our understanding of how macro-level forces influenced ART users' beliefs and expectations and subsequent sexual behaviours and outcomes. We attended to the way in which gender and stage of ART use and regained health might have a bearing on the participants' sexual desires and behaviours. After the several iterations of analysis, we settled on a final set of conceptual categories: "ART users attitudes towards sex", "normalization of health and increased sexual desire", "optimism about the future", "difficulty finding a sexual partner and disclosure of HIV serostatus".

## Results

### Socio-demographic characteristics of participants

The ages of the participants ranged from 22 to 62 years. The median age was 35 years for women and 40 years for the men. Most of the women (18 out of 20) were widowed compared to men almost half of whom reported this status (11 out of the 21). Four women and one man were Moslems while the rest were Christians (Catholics, Protestants, Pentecostal and very few seventh day Adventists). Socio-economic status was poor, consistent with levels of poverty in the general population. Only five men and one woman ever held a salaried job, the rest were subsistence farmers, small scale traders or manual traders.

### "Those things are not in my heart" - ART users' attitudes towards sex

Many participants reported that their sexual function had been affected by HIV illness. They talked about their sexual desires having changed over time with many reporting diminished desire at three and six months on ART compared to 18 and 30 months of use.

#### Reasons for remaining abstinent

There were varied reasons as to why many participants had remained abstinent despite reporting that their health had improved since starting ART. Some of their reasons revolved around misconceptions and fears of the consequences of engaging in sex on health. Both women and men reported similar reasons for having not resumed sex and these were: not having recovered fully, insufficient energy and lack of privacy as they shared a sleeping house with children, lost interest in sex, followed the health workers advice of not engaging in sex, fear of superinfection, fear that the virus would regain strength, did not trust their partners and that they no longer considered sex as an important part of their lives.

At three months on ART, many still complained of general body weakness and recounted the effects of the illness on their lives, including their physical appearance and sexual function. They were more concerned about improving their health than engaging in sex.

I abstained from sex and no longer involve in it. Even the people tell me that I did right to give up on such things for I would be dead by now. [FFH5-3 months]

Since I joined TASO I lost appetite [interest] about having sex I have never had sex again. [MFL3-3 months]

A man who said he feared superinfection and did not have sufficient energy to engage in sex said:

The one you expect not to have it [the virus] will have it and you just get another one [new virus] ...I also haven't got that energy yet. [MFH4-3 months]

However at three months many men were more optimistic about resuming sex sooner than women. Two men talked about this in the following excerpts:

I: Does it mean that you lost desire for it [sex]?

P: No, I still have desire but I still want to gain more health. If God wishes I will get a partner. [MFL3-3 months]

A second one said:

*...I told you that I stopped that [sex] but you never know if God recovers me well, I will get one ... I want to get a person who knows my problem and whom I know her problem so that we can remind each other to take the drugs. [MHL4- 3 months]*.

Concerns about the interaction between sexual activity and ART were common. Many women believed that ART had made the virus dormant and thus resumption of sexual activity would awaken the virus.

One woman said:

With my sickness when I engage in sex, I can make the virus regain strength. [FFL5-3 months]

Some of the male participants reported that the experience of knowing that they were HIV-seropositive was very threatening.

As I told you that I was very much scared when I knew I had AIDS, I lost interest up to now I am not yet ready for sex. I am too scared. Although I was given condoms, I do not practice it. Imagine from November 2005 up to now I don't know how my wife looks like (no sex for the last 6 months). [MFL1-3 months, married]

At three months on ART many participants still had fresh memories of the consequences of HIV on their lives. At this stage, many were clearly not in favour of sexual activity and reported that their experience with illness had taught them that sex was not something to be rushed into but to be approached more carefully. In addition to their concerns, they were still physically weak and had lots of physical signs of the illness. They talked about their desire to advise partners to go for tests before they decided to have sex with them and also using condoms with partners all the time. This was however, not the case for many of them as they improved health and engaged in sexual relationships without disclosing their own status or knowing the HIV status of their partners.

At 6 months on ART, almost all the women interviewed (11/14) and some of the men (9/20) still reported abstinence due to the same fears they had reported at three months on ART.

Ever since I started taking these drugs, I have never had sex again...I no longer like sexual relationships because of what happened to me; I gave up all about sex. [MFL1-6 months]

Some of the participants who had started engaging in sex, had concerns about pregnancy. Many had children prior to falling ill and some had been forced to send their children to live with relatives when they were unable to take care of them. They also talked about their fears of bearing children who were HIV positive and the burden they would leave their relatives taking care of their children and therefore felt that having any more children was not a good idea:

I have taken a long time without having sex I am still seeking for health I can't engage myself in such issues now. I told you that I have children and I don't stay with them. Now if I get a partner she has to deliver more children and it will be a problem again on my side. [MFL3- 6 months]

It is interesting to note that even at 18 and 30 months on ART a few of the participants still had the same fears they had when they were interviewed at 3 and 6 months on ART. Some still talked about sex as something that 'hurt' their health and should be avoided while others reported that they still had vivid memories of the counselling sessions they had received concerning sexual activity. They also talked about being focused on bringing up their children now that they were feeling better while others reported a diminished sexual desire as a result of ageing.

I do not think I will change [decision to abstain] because I don't have it in mind...this is because of the rules attached to the medicine, they do not recommend it. For the sake of my health, I cannot involve myself in that ...It is something that hurt my health that is why for me I decided to give up on that. [FHL4-30 months on ART]

I don't have such energy, the little that I remained with can't be shared and I still remain with life; ...Ah aha, according to what I have seen I don't still have that time, the things that I am thinking are very different ...I am thinking about what my children are going to eat at lunch. [MFL2-30 months]

### Normalization of health and progression of sexual desire over time

Although some participants had chosen to remain abstinent, others had resumed sexual activity at different stages of ART. According to many participants their desire for sex was reversed by ART and hence with continued use, they reported that their desire had increased markedly. While women were likely to mention their motivations to resume sexual activity as being desire for children and material support, men mentioned reasons such as: for companionship and wanting someone to take care of their physical needs such as cooking, social norms about having a spouse, and needing someone to satisfy their renewed sexual desires.

Compared to women (5/20), relatively more men (8/21) reported having resumed sex at three months. They said that their main reason for resuming sex at three months on ART was mainly for the sake of their partners. A male participant separated with his wife:

For me I didn't have energy for having sex but I could force myself in order not to lose the marriage. Now that I am alone, I am just thinking about my life. [MHH5-3 months]

Participants expressed a desire to find partners of a similar HIV serostatus and also on ART. They said that they hoped that in addition to catering for their sexual needs, such partners would provide them with support in taking medications as they too would have experienced what it is to be on ART. They talked about finding such partners through the HIV clinics they attended:

I told you that I stopped that and I sealed it but you never know if God recovers me well, maybe I will get one [partner] but I must get one who is already infected. I will go to the office and enquire from my counsellor whether there is a person who is HIV positive like me. I want to get a person who knows my problem and I know her problem so that we can remind each other to take the drugs. [MHL4-3 months]

At six months on ART, half the men (10) reported that they had resumed sexual activity but mainly with their wives. They attributed their renewed sexual desire to the use of the ART and a sign that their health was improving.

I am regaining the desire for sex unlike before when I had no interest in having it...that shows my health is improving. It is not like before when I had lost appetite and I couldn't eat anything. [MHH4, 6 months]

Participants had now started talking about sex as a natural thing and for those who said that they had not resumed sex acknowledged that they were now developing interest. Given that many of the participants were unmarried (18/20 women, 11/21 men at enrolment), many were thinking about the possibility of long-term relationships and marriage.

At 18 months, some of the participants especially men who reported that they had resumed sex had started arguing that sex was something made by God and should not be neglected. Although many of the participants reported that they had resumed sex, their sexual encounters were less frequent compared to before they fell ill and started ART.

Well, that one is not compulsory that I have to be with a sexual partner but as you know things which were made by God [meaning things like sex] you cannot reject because of having AIDS. When I feel like having it [sex], I can get one but not frequenting it...I don't deny I get one. [MFL3- 18 months]

It was observed that many of the women who had mentioned in the interviews at three and six months that they would never engage in sex were now changing their views on this allowing for the possibility of engaging in sex.

My friend as I told you that you never know, it is what happened to me when I took these drugs, my life improved and even the desire for sex restarted and I got a sexual partner and I found myself being pregnant. [FFL3-18 months]

Although many participants had resumed sexual activity at 18 months on ART, they acknowledged the challenges of 'safer sex'.

...Love is blind and it is worse with alcohol, you can suspect someone to be HIV positive before taking it [alcohol] and after taking it you become blind. After having sex for sometime the mind forgets all about the sickness and you remove the condom. [MFL4- 18 months]

Participants described health as being synonymous to 'life'. They recounted what they had reported in the interviews in the earlier rounds about never engaging in sex, but as their health got better it became difficult to keep their word. Hence, at 30 months on ART, many participants considered themselves as "normal" and said that as "normal people" one of the healthy functions was to have sexual desires. They also reported that drugs had increased their desire for sex, contrary to what many had mentioned at three and six months about the drugs having diminished their sexual desire:

...After taking drugs for six months the desire started. It was about 50%. [...]My life had returned, I had strength, each of my joints was so fit. [MFL4 - 30 months]

I know I had promised you [interviewer] that I would never have a partner, but here I am with one already. Let me tell you [interviewer], "when there is health there is life". I had thought that I would never work. These thoughts were similar to ever having a relationship. I tried very much to be single but when I couldn't hold on any longer I got someone who was also with TASO and is on medication. [FFL5 - 30 months]

### Finding a sexual partner, serosorting and disclosure of HIV status

At six and 18 months on ART, those who reported that they had a renewed desire for sex said they found it difficult to find sexual partners from their villages as many people knew their HIV status. Many mentioned that they had managed to get sexual partners (some also HIV positive) through their peers (also on ART). They reported that they had requested the counsellor to help them to find a suitable person

*God helped me [...] I got a partner and she is also HIV positive and I got her from TASO. The problem was solved and I am now okay*. [MHH5- 18 months]

Since many women encountered difficulty finding sexual partners, they reported handling their relationships discreetly so that other people who knew their status did not know about it as they could disclose their status to their partner. Some had plans of getting married to their partners but feared disclosing their HIV serostatus as this could risk being abandoned. One woman who had thought she would never find a partner talked about her experience at 18 months on ART as follows:

It is difficult to get a partner unless he is also of the same status. What helped me was that my partner came here [village] to work [...] for me to see him, he came here to make a call when his phone battery was low. He continued coming here to make calls and that is when our relationship started. I think he had already developed an interest in me...I am known to be HIV positive and if I stand with a man who is not my relative and people see me, they quickly warn him about me being infected. They must warn him [...] he doesn't know that I am HIV positive and that I am on drugs so if I get married to him how would I manage to go and collect my drugs from TASO?[...]My question was that how would I collect my drugs and how would I swallow them if I stayed with him, he should have questioned himself why am I taking drugs daily? [FFL3- 18 months]

Some women favoured male visitors to their villages. Most of them also reported that they did not talk about their HIV status with new partners. They however, expected other people to inform their partners:

I didn't know his status but what I think is that he knows about my health status because the people he stays with at that home know very well about my status. Sometimes the motorcycles with a TASO label find him here. This shows that it comes from TASO. [FHL5-30 months]

One of the reasons many participants mentioned for desiring to have a partner who was HIV seropostive like them was because this would help them with their drug adherence. They reported that with such partners they did not need to take their medications discretely:

...when I looked at this one, she falls in my category so I said that it would help me not to go out for more partners because that would cause me problems. This is because if you go out with someone that doesn't know your status or who knows nothing about these things you will start hiding the tablets from her not to see you swallowing them. [MFH2-30 months]

Similarly, while many men reported that they would like to have partners of a similar HIV serostatus to their own, when they commenced sexual relationships, they rarely enquired about the HIV serostatus of their partners. Some participants thought that so long as they were protecting their partners from HIV infection by using condoms, they did not see the need to disclose their HIV status to them.

Moreover, men interpreted that their partners agreeing to have sex with them meant that they were also HIV positive and hence did not need to disclose their HIV status to their partners when they first met:

That lady is a resident of this village and she very well knows me because she has seen me for a long time. By the time I fell seriously sick, she used to come and pay me visits... I think she might also be HIV positive because I fell sick and everybody knew that I was suffering from AIDS. She was among those paying me visits and saw how I suffered but when I asked her for love she didn't hesitate which indicated to me that she might also be HIV positive. [MFL4-6 months]

At 18 and 30 months on ART men talked about their physical appearance having normalized as they no longer have any physical signs indicating that they were HIV-sero-positive. Whenever they approached women, they could easily consent to their sexual advances without asking them about their HIV serostatus.

...It is not hard to find a partner... because I do not have any signs that may scare a person. [MHH4-18 months]

I had taken long without a partner ever since I lost my wife... These days I am a nice looking guy and admirable, whoever I talk to has to accept [agree to his sexual proposal]...I started having sex and I have asked the doctor to provide me condoms. I can't hide it from you, I have a partner now. [MHL4 - 30 months]

It was interesting to note that for some women, being on ART for a period of 30 months seemed to have increased the autonomy in selecting partners who would provide for their material as well as sexual needs and would agree to use condoms. Several women reported that although they had now agreed to have sex with fellow HIV positive people after refusing for some time, the relationship was mainly shaped by their wishes.

He [new partner] had to promise me that he would abide by my terms and conditions of love using condoms all the time and avoiding pregnancies and any other sexual diseases. [FFL5 - 30 months]

### Hope for the future

Availability of ART was understood as providing hope and an increased ability to plan for the things that would return life to normal. Among some of these things were having a spouse and children and material property such as a house. In as much as resumption of sexual activity could help meet some of the desired goals such as having a companion and for a few, children, some talked about it as a way to achieve developments such as affording to build a house or revive their petty trade that had collapsed when they succumbed to illness.

...I had just come back from the island and I had worries, they had burnt down my fishing net, my money had got finished. So my uncle told me to go back home. "With the worries you have now, you can even drown in the lake". I then came back. At that time, in order to have a rest from my worries, I would most of the time be taking alcohol. And I was taking too much booze. Ha, and it's at that time that he proposed to me. I first refused but I then later said but I have a lot of worries! Maybe this man would give me a rest from the worries. Ha, we then fell in love and he told me to look for a house and he paid the rent. [FHH3 - 30 months]

While some women talked about being motivated to find partners to cater for their difficult financial situation and hence freeing them from depending on relatives, others, especially those who had material property, talked about avoiding forming partnerships for fear of losing their material possessions. At 18 and 30 months on ART, most of the participants reported that they would like to have partners who were interested in marriage rather than just sex.

*I am now looking for a partner to get married to because I have a house...I don't want someone to give me a headache, I need a person from TASO who is aware of my status and I also know hers*. [MFL4- 18 months]

### ART users' efforts towards HIV prevention and health promotion

Nine participants (7 women, 2 men) aged between 30 and 50 reported that they had abstained throughout the study period. Fear of infecting one's spouse was common among the participants who reported their spouses to be HIV sero-negative. A married man with a sero-discordant wife talked about his experience in the following:

*What should I hide from you my sister, the sexual desire came back and we had sex for sometime but recently my wife refused completely saying that I want to kill her leaving the children alone. Then I got stress for that action because I was strong and my wife never wanted me to disturb her [have sex with her]. She says that though we use condoms, she doesn't have the appetite [interest] for sex. We got some misunderstandings and she went to report me to our counsellor, so I also gave up with it [sex]... She says that if possible I can get another person but for me I don't like to get a new one. [MFL1 - 30 month*]

Due to the concern some of the male participants had about infecting their sexual partners with HIV, they reported that they had decided to use condoms even though inconsistently and usually only at the start of new relationships. Many had also a previous dislike for them.

... Although TASO gave us seminars about condom use, I couldn't buy that idea. All that time, which I have spent on drugs, I have not had that feeling [sexual desire] until recently when I started getting it. Since I have it now, we have to use condoms in order not to infect ourselves... [MHL4-30 months]

While people reported marked sexual desires at 30 months on ART; they also indicated having reduced their number of partners and instead desired partners who were faithful.

... I remember once I told you that I had got a partner but I failed to continue with her because she had many sexual partners. Fearing that I might acquire other viruses which I didn't have, I left her and got this one so that I also get that enjoyment. [MFL4- 30 months]

## Discussion

The findings have indicated that as people's health improves due to continued use of ART, many have renewed sexual desires and may resume sexual activity for reasons such as: companionship, to save their marriages, to have children, material support and to satisfy their sexual desires. Some may engage in unprotected sex despite counselling and availability of condoms. Similar findings have been noted by others pointing to many people diagnosed with HIV claiming to have a loss of sexual interest and their plan to remain abstinent at ART initiation [14]. As observed by Seeley et al., [11], establishing relationships and fulfilling one's sexual desires is something that ART has bestowed on many. Our findings show that as many people regained their health due to continued use of ART, they resumed sexual activity usually with new partners.

Although many participants reported reduced numbers of partners, the ART users in this study found it challenging to consistently adopt safer sexual behaviours such as using condoms. As noted in other studies [15], a new cadre of patients is emerging-'those who expected to die but are now getting better'. With renewed health and hopes, this group may facilitate the further spread of the epidemic or control it. As also observed elsewhere [8], counselling and free condoms, did not lead to long-term consistent condom use. Therefore, knowledge about HIV and how to avoid the disease does not automatically translate into less risky sexual behaviours and much more prevention efforts might be necessary.

It was encouraging to note, that ART use seemed to have empowered some women to discuss their sexual and reproductive health issues such as condom use and sometimes their fertility intentions with their partners. The motivation behind all this was to preserve their health after experiencing severe illness and near death signs that they could not believe that they would ever get better.

As many people regain health and report increased sexual desire, it is a challenge for couples to consistently use condoms. Besides, in relationships where the women totally refused sex for fear of infection, their partners may engage in extramarital sex in a bid to satisfy their desires and in the course place other people at risk. The challenge therefore, is how we can ensure that people on ART satisfy their sexual desires and other sexual and reproductive needs, one of their fundamental human rights [16] but at the same time ensuring that their behaviour does not place them and others at risk.

Participants talked about finding partners who were also HIV positive like themselves. Their partner selection strategies were mainly motivated by their desire to find someone who could support them in their drug taking but also driven by a desire not to infect others. While many participants' desire to have partners of the same HIV status was encouraging, it is important to point that while in theory selecting sex partners of the same HIV status could offer protection against transmission, in practice the value of serosorting may be questionable [17]. Since the HIV serostatus of partners was often assumed rather than openly discussed, this might mean that many of the partners were placed at risk. Eaton [17] observed that for the HIV positive persons who can be certain about their HIV status, serosorting can provide benefits. However, unprotected sex between HIV sero-positive persons carries risks such as superinfection, sexually transmitted infections and unplanned pregnancy that should be considered when making sexual and reproductive health decisions. It is apparent that many couples should be encouraged to go for frequent testing to ascertain their status and that of their partners instead of assuming that they might be infected because they once had a sexual relationship with someone who had been tested and found to be HIV-sero-positive. It is also crucial for interventions to focus their messages on the potential risks of having unprotected sex, even with someone of a similar HIV status.

It was evident that people made efforts to change sexual behaviours after they started ART and realised that their health was improving. Participants reported their decisions to adopt safer sexual behaviour such as reducing number of partners, use of condoms, abstaining and remaining faithful to their spouses. The fear of superinfection was certainly a concern for many participants and may be interpreted as beneficial in some ways. In as much as participants would like to satisfy their increasing sexual desire, they are cautious about the risk this might cause to their improving health. In the course of being cautious about their health, it can be argued that they are also protecting other people. In that view, ART can be regarded as having long-term beneficial effects on HIV incidence and hence provide a motivation for further scale up. This finding concurs with what has been reported in other studies which have noted that abstinence and reduced sexual risk behaviours to be preventive strategies adopted by HIV-seropositive persons aware of their serostatus [6,18]. It is also becoming apparent that good adherence to ART and therefore viral suppression could help in prevention of infections [19,20]. Continued counselling after ART initiation could be useful in wielding social control over risky sexual behaviours and as a result, manage the spread of HIV/AIDS.

Since many of the participants especially women were unmarried, they talked about difficulty finding sexual partners. As some individuals experienced difficulties they resorted to changing residence and going to areas where no one knew their status, and not disclosing their status to new partners for fear of being abandoned. This finding compares with what was found in other studies [1,2,21,22] conducted in Uganda. They noted that due to societal expectations, many individuals may not disclose HIV serostatus in sexual relationships. It is apparent that the improving desire for sex due to continued use of ART and the difficulty of finding a sexual partner has implications for sexual risk and HIV spread. We observed in our study that as many people regain health and their sexual desires are awakened, they are likely to migrate to new areas so that they can be able to find new partners who have little information about their sexual health history. This may be risky as it may encourage the spread of the disease.

It was of concern that many of the individuals, who reported renewed sexual desire and subsequently engagement in sexual activity, did not sometimes know the HIV serostatus of their partners. Many reported that they expected their partners to know that they were HIV-seropositive sometimes through gossip from other people who had seen them when they were seriously ill. This finding is similar to what was observed in other parts of Uganda [2,3] where, many cohabiting partners were noted not to mutually know their HIV status. An interesting thing to note is that although many individuals reported that they would have liked to have a partner of a similar HIV status, in practice many did not know the HIV serostatus of their partners.

The fear that existed among women about losing their partners if they knew their HIV status by seeing them take medications, is evidence of the stigma that still exists and how this was a barrier to disclosure of one's HIV status. As much as it has been assumed that access to ART could reduce stigma and subsequently enhance disclosure, this may not be straightforward. As noted by Makoae et al. [23], taking medications daily may indicate that someone is HIV-positive, and therefore someone who does not wish to disclose status may avoid taking medication. It has also been observed that disclosure of HIV status is often associated with a marked anxiety and fears of rejection [10], which in itself hinders safer sex. This may indicate that as people's health improves and their desire for sex is heightened, they are likely to prioritise non-disclosure relative to disclosure, so as to overcome the barrier of finding a sexual partner to satisfy their sexual desires and other needs.

Although many individuals reported an increased desire for sex over time, they also talked about a wish to use condoms, have fewer partners and to avoid child bearing. However, as they continued taking ART (18 months and 30 months), many found it difficult to use condoms consistently and to prevent pregnancy. These findings are in keeping with what was observed in other African settings, which pointed to some risky behaviours decreasing with ART use while a substantial proportion continued to have unsafe sex [10,24]. Disclosure and couple counselling alongside mutual support may play a central role in sustaining safer sex. It is however, important to point out that continued efforts on HIV prevention (through safer sex) should also be focused at the wider community and not just on the ART users. It can be argued that as many ART users regain their health, reminding others of their status (disclosure) would be of low priority since it removes the "healthy tag" that they very much craved for and are now gradually achieving. Expecting the ART users to be on the forefront of prevention behaviour although a good idea, it could also be interpreted as over burdening them. As indicated in the findings, many ART users held the view that anybody who did not insist on condoms and easily consented to their sexual advances must have also been HIV-sero-positive.

Conducting longitudinal interviews at four different time periods of ART use, allowed for the understanding of how people's feelings and desires for sex progresses over time with renewed health but also allowed for the clarification and deeper understanding to be gained concerning sensitive issues. Longitudinal qualitative research though underutilized in medical research is becoming increasingly valuable [25]. Despite the rigour as indicated in the longitudinal qualitative approaches utilised, this study has some limitations. The study was conducted among a carefully monitored cohort that received frequent counselling and medication through a HIV programme -TASO and hence we are not certain whether similar results may come up from a non-programme setting. Besides, we are unsure of whether the reported improvements in health would continue along the same trends over time thus presumably affecting their sexual desires and behaviours over time. It would be interesting to conduct similar interviews probably five years or beyond on ART to see whether some of the reported sexual desires and behaviours have changed-positively or negatively. Reflecting on the role of the researcher on the research, different people performed different roles (i.e. the trial team was separate from the qualitative data collection) and hence minimising researcher/intervener bias.

## Conclusion

As the health of people living with HIV improves due to continued use of ART, many resume sexual activity. For some people in our study this was after a period of abstinence. A number of participants reported a reduced number of partners and being faithful to their spouses or permanent partners. This in itself is encouraging and a further drive to increase ART access for people living with HIV. The challenge however, was their ability to use condoms consistently, their desires to fulfil cultural expectations around having a family (marriage and children) and the difficulty of finding sexual partners for those who would like to engage in sex. These challenges, particularly the difficulty of finding a sexual partner, have implications for adherence to ART and disclosure of HIV serostatus. They may encourage the spread of HIV to new areas where people living with HIV might move to with a hope of finding new sexual partners who do not know their HIV status especially if this leads to poor adherence.

These findings point to the need to ensure that counselling messages reflect the changing realities of the ART users. There is also a need to intensify counselling efforts so that those on ART feel comfortable disclosing their HIV status to sexual partners and practice safer sexual practices. Moreover, counselling message should reflect the changes in perceptions and priorities for ART users so that as health returns these people are assisted to make safe and informed decisions. But prevention should not be restricted to people living with HIV alone, the general public needs more encouragement to test, know and act responsibly.

## Competing interests

The authors declare that they have no competing interests.

## Authors' contributions

JW analysed and wrote the first draft of the paper. MM coordinated the field work and contributed to the analysis and write-up. JS provided technical advice on the study design, analysis and interpretation of data and write-up of the paper. JB helped in implementing the ART programme and coordinate TASO activities. SJ participated in the overall design of the main trial study and provided technical support on the data collection. All authors read and approved the final manuscript.

## Pre-publication history

The pre-publication history for this paper can be accessed here:

http://www.biomedcentral.com/1471-2458/11/633/prepub
